# Clinical, phenotypic and oncologic background differences in patients with sporadic, familial and multiple primary melanoma^؆^

**DOI:** 10.1016/j.abd.2026.501472

**Published:** 2026-10-31

**Authors:** Irene López-Riquelme, Silvestre Martínez-García, Marina Romero-Bravo, Miguel-Ángel Berciano-Guerrero

**Affiliations:** a Department of Dermatology, Hospital Regional Universitario de Málaga, Málaga, Spain; b Department of Oncology, Hospital Regional Universitario de Málaga, Málaga, Spain; c Instituto de Investigación Biomédica de Málaga-Plataforma BioNAND, Málaga, Spain; d Department of Medicine, Universidad de Málaga, Málaga, Spain

**Keywords:** Genetic predisposition, Melanoma, Risk factors, Phenotype, Solar radiation

## Abstract

**Background:**

Sporadic Melanoma (SM) refers to patients with a single melanoma and no family history. Multiple Primary Melanoma (MPM) includes individuals with two or more primary melanomas. Familial Melanoma (FM) is defined as melanoma occurring in two first-degree relatives, or in three or more affected family members of any degree. Previous studies describe distinct phenotypic and clinical profiles in each subgroup, and a high proportion of personal and familial cancer history in patients with melanoma.

**Objective:**

To compare phenotype, clinicopathological features, and oncological history in the three groups.

**Methods:**

Data from 442 patients were collected and divided into three groups: FM, MPM, and SM.

**Results:**

The MPM group showed light eye color (p = 0.01), more tan 50 nevi (p = 0.00014), actinic keratoses (p = 0.0025), and melanomas arising from pre-existing nevi (p = 0.0031). FM patients were younger (p = 0.01) and had a higher burden of personal and family cancer history, particularly breast neoplasms (p = 0.01). SM presented ≥ 2 mitoses (p ≤ 0.0001). Multivariate analysis confirmed independent associations specific to each group.

**Study limitations:**

Long term-follow-up for secondary melanoma is needed for validation. Also, some participants may not recall certain family history.

**Conclusion:**

Patients with MPM display fair phototypes, numerous nevi, and markers of chronic sun damage, suggesting a central role of phenotype and cumulative UV exposure. In FM, younger age and a higher burden of oncologic history point toward an underlying genetic predisposition. SM is characterized by fewer nevi and aggressive melanomas, reflecting a distinct biological entity. These findings highlight the complex interplay between genetic and environmental factors.

## Introduction

Cutaneous melanoma has been increasing in incidence in recent years. Its pathogenesis involves a complex combination of environmental factors and individual genetic susceptibility. The main clinical and epidemiological factors are the presence of numerous melanocytic and atypical nevi, low phototype, chronic sun exposure, and a family history of melanoma.^1^

The first genes linked to melanoma were CDKN2A and CDK4, which are also associated with pancreatic cancer. In subsequent years, other genes have been described, mostly tumor suppressor genes (such as MC1R, MITF, POT1, TERT, ACD, TERF2IP, PTEN, BRCA2, BRCA1, RB1, NF1, and TP53), associated with internal malignancies.^2^

Based on family history and the number of melanomas, patients are classified:

• Sporadic Melanoma (SM): refers to patients with a single melanoma and no family history. It accounts for 90% of cases.^3^
• Multiple Primary Melanoma (MPM): this term comprises individuals with two or more melanomas, synchronous or metachronous, without a family history. Its prevalence is estimated at around 5%‒10% of cases.^3^
• Familial Melanoma (FM): refers to patients with melanoma and two first-degree relatives affected, with or without identified mutations.^4^ Less strict definitions include second-degree relatives or even three or more relatives of any degree, although this is not clearly defined in the literature. They frequently develop multiple melanomas. This group account for between 5% and 10% of all melanomas.^5^
• Hereditary Melanoma (HM): this term is reserved exclusively for melanoma patients with mutations in high-penetrance genes. Some studies use the term “hereditary” more broadly to also include carriers of low- or moderate-penetrance mutations that increase risk but do not follow a clear Mendelian pattern (polymorphisms in MC1R, MITF, TYR, OCA2, or SLC45A2).^2^, ^6^, ^7^ Approximately 20% of patients with familial melanoma are included in this subgroup (2% of all melanomas).^8^ Currently, although the availability of genetic tests varies between countries, in most regions it is indicated to perform genetic studies in patients with familial melanoma and multiple primary melanoma.^9^

Patients with FM present at younger ages and more frequently develop multiple melanomas.^10^ Nevertheless, individual risk is determined by the specific gene involved, together with environmental exposures and clinical and histopathological characteristics. Grange et al., in 1995, observed a higher risk of FM in red-haired individuals with atypical nevi.^11^ In 2008, Nagore et al. linked familial aggregation to atypical nevi, multiple melanomas, and the absence of perilesional sun damage.^12^ Subsequently, these characteristics have been associated to mutations in CDKN2A and MC1R,^6^ as well as histopathological patterns (thickness, infiltrate, regression, pagetoid invasion, atypia, mitotic index). In this regard, in 2014, Aguilera et al. associated CDKN2A mutations with a lower Breslow thickness and a higher rate of melanoma in situ.^13^

Recently, a high burden of personal and family history of cancer has been observed in FM and MPM.^14^, ^15^ This has led to the proposal of broader criteria for genetic testing (for example, any history of neoplasia or age of presentation less than 35 years).^16^

To date, no studies have directly compared these three groups. Therefore, the authors aimed to evaluate differences in age at presentation, phenotype, clinicopathological features, and oncological history among patients with FM, MPM, and SM. Improved characterization of these groups may facilitate the identification of patients with familial predisposition, support preventive strategies, and help define prognostic factors.

## Methods

A retrospective, cross-sectional observational study was designed, including patients diagnosed with melanoma at the Dermatology Department of the Regional University Hospital of Málaga from 2000 to 2025. Data collection was carried out during their follow-up visits between January 2023 and February 2025. Patients with uveal and mucosal melanoma or melanoma in locations other than skin, as well as metastatic melanoma without a known primary tumor, were excluded. Patients followed for a known germline mutation or family history who had not developed melanoma were also excluded.

Epidemiological, clinical, and histological data were collected, and patients were questioned about their personal and family history of cancer. The number of patients was considered (not the number of melanomas). In the case of patients who had presented with multiple melanomas, the clinical and histological data of the first melanoma were collected.

Three groups of patients were established:

1) SM: patients with a single melanoma, without family history of melanoma or pancreatic cancer.
2) MPM: patients with two or more melanomas, without family history of melanoma or pancreatic cancer.
3) FM: patients with melanoma and at least one 1st or 2nd degree relative with melanoma and/or pancreatic cancer, or three or more relatives of any degree in the same family.

### Statistical analysis

This study follows the STROBE guidelines for observational studies (see Supplementary Material: STROBE Checklist). The statistical software RStudio 2024.12.1 was used. A descriptive study of the variables was conducted, calculating absolute and relative frequencies for qualitative variables and arithmetic mean, standard deviation, minimum and maximum values for quantitative variables. The 95% confidence interval was estimated.

For bivariate analysis, appropriate parametric and non-parametric tests were used. For comparisons of categorical variables, the Chi-Square test and Fisher's exact test were performed, depending on the expected frequencies. All tests were two-sided, and *p* < 0.05 was considered significant. In multivariate analysis, *p* < 0.2 was considered indicative of a trend toward significance due to its potential clinical relevance.

### Ethical and legal aspects

The study was approved by the Provincial Research Ethics Committee of Málaga and was conducted in accordance with the Declaration of Helsinki and Good Clinical Practice guidelines. The processing of personal data complied with Regulation (EU) 2016/679 and Organic Law 3/2018 on data protection.

## Results

A total of 442 patients were included: 195 men and 247 women, with a mean age of 51.7 ± 15.3 years (95% CI 50.3–53.1). They were divided into three groups: SM (*n* = 263), FM (*n* = 132) and MPM (*n* = 47). Clinical, phenotypic and histopathological characteristics were compared between the groups, as shown in Table 1.

**Table 1:** Distribution of the epidemiological, clinical, and histological characteristics of the patients, with p-values < 0.05 considered statistically significant and shown in bold.

Variables			Sporadic melanoma	Familial melanoma	Multiple primary melanoma	Total	*p*
			(*n* = 263)	(*n* = 132)	(*n* = 47)	(*n* = 442)	
**Phenotypic characteristics**	Sex	Male	117 (44.5%)	49 (37.1%)	29 (61.7%)	195 (44.1%)	**0.01**
		Women	146 (55.5%)	83 (62.9%)	18 (38.3%)	247 (55.9%)	
	Age (years)	<45	76 (29%)	58 (43.9%)	14 (29.8%)	148 (33.6%)	**0.01**
		≥45	186 (71%)	74 (56.1%)	33 (70.2%)	293 (66.4%)	
	Phototype	I-II	193 (73.4%)	100 (75.8%)	41 (87.2%)	334 (75.6%)	0.13
		III-IV	70 (26.6%)	32 (24.2%)	6 (12.8%)	108 (24.4%)	
	Eye color	Light	95 (36.1%)	38 (28.8%)	25 (53.2%)	158 (35.8%)	**0.01**
		Dark	168 (63.9%)	94 (71.2%)	22 (46.8%)	284 (64.2%)	
	Hair color	Light	71 (27%)	37 (28%)	21 (44.7%)	129 (29.2%)	**0.05**
		Dark	192 (73%)	95 (72%)	26 (55.3%)	313 (70.8%)	
	Number of nevi	< 50	193 (73.4%)	80 (60.6%)	21 (44.7%)	294 (66.5%)	**0.00014**
		≥ 50	70 (26.6%)	52 (39.4%)	26 (55.3%)	148 (33.5%)	
	Solar lentigines	No	73 (27.8%)	44 (33.3%)	15 (31.9%)	132 (29.9%)	0.49
		Yes	190 (72.2%)	88 (66.7%)	32 (68.1%)	310 (70.1%)	
	History of sunburns	No	49 (18.6%)	23 (17.4%)	7 (14.9%)	79 (17.9%)	0.82
		Yes	214 (81.4%)	109 (82.6%)	40 (85.1%)	363 (82.1%)	
	Actinic keratoses	No	191 (72.6%)	98 (74.2%)	23 (48.9%)	312 (70.6%)	**0.0025**
		Yes	72 (27.4%)	34 (25.8%)	24 (51.1%)	130 (29.4%)	
	NMSC	No	213 (81%)	100 (75.8%)	29 (61.7%)	342 (77.4%)	**0.01**
		Yes	50 (19%)	32 (24.2%)	18 (38.3%)	100 (22.6%)	
**Clinicopathological characteristics of melanoma**	Location	Abdomen	15 (5.7%)	9 (6.8%)	3 (6.5%)	27 (6.1%)	0.82
		Head and neck	33 (12.6%)	13 (9.8%)	7 (15.2%)	53 (12%)	
		Neckline	15 (5.7%)	11 (8.3%)	4 (8.7%)	30 (6.8%)	
		Back	91 (34.6%)	53 (40.1%)	18 (39.1%)	162 (36.7%)	
		Lower limb	57 (21.7%)	21 (15.9%)	7 (15.2%)	85 (19.3%)	
		Upper limb	52 (19.8%)	25 (18.9%)	7 (15.2%)	84 (19.1%)	
	Subtype	Unknown	1 (0.4%)	2 (1.5%)	0 (0.0%)	3 (0.7%)	0.30
		Superficial spreading	188 (71.5%)	106 (80.3%)	40 (85.1%)	334 (75.6%)	
		Acral lentiginous	10 (3.8%)	2 (1.5%)	1 (2.1%)	13 (2.9%)	
		Lentigo maligna	24 (9.1%)	8 (6.1%)	1 (2.1%)	33 (7.5%)	
		Nodular	40 (15.2%)	14 (10.6%)	5 (10.6%)	59 (13.3%)	
	Breslow	<0.8 mm	127 (48.7%)	78 (60.9%)	27 (57.5%)	232 (53.2%)	0.06
		≥0.8 mm	134 (51.3%)	50 (39.1%)	20 (42.5%)	204 (46.8%)	
	Ulceration	No	228 (88.7%)	119 (96%)	40 (90.9%)	387 (91.1%)	0.07
		Yes	29 (11.3%)	5 (4%)	4 (9.1%)	38 (8.9%)	
	Number of mitoses	0‒1	133 (55.4%)	87 (73.1%)	36 (83.7%)	256 (63.7%)	**< 0.0001**
		≥2	107 (44.6%)	32 (26.9%)	7 (16.3%)	146 (36.3%)	
	Origin	De novo	218 (85.8%)	98 (80.3%)	30 (65.2%)	346 (82%)	**0.0031**
		Pre-existing nevus	36 (14.2%)	24 (19.7%)	16 (34.8%)	76 (18%)	
	Inflammation	Unkown	27 (10.3%)	11 (8.3%)	6 (12.8%)	44 (10%)	**0.04**
		Absent-mild	188 (71.8%)	103 (78%)	34 (72.3%)	325 (73.7%)	
		Moderate-intense	47 (17.9%)	18 (13.7%)	7 (14.9%)	72 (16.4%)	
	Metastasis	No	218 (82.9%)	116 (87.9%)	43 (91.5%)	377 (85.3%)	0.19
		Yes	45 (17.1%)	16 (12.1%)	4 (8.5%)	65 (14.7%)	

### Epidemiological and phenotypic characteristics

The authors observed a male predominance (61.7%) in the MPM group, compared to 44.5% in the SM group, and 37.1% in the FM group (*p* = 0.01). Regarding age, the authors found a higher percentage of patients under 45-years in the FM group (43.9%) compared to the other two groups (29% in SM; 29.8% in MPM) (*p* = 0.01).

In the MPM group, 53.2% of patients had light-colored eyes. However, only 31.6% of the SM group and 28.8% of the FM group presented this characteristic (*p* = 0.01). Similarly, 44.7% of patients in the MPM group were blond or red-haired, while only 27% of the SM group and 28% of the FM group presented this characteristic (*p* = 0.05).

On the other hand, it is striking that in the MPM group, 55.3% had a melanocytic nevus count greater than 50, while in the SM and FM groups, only 26.6% and 39.4%, respectively, had more than 50 nevi (*p* = 0.00014). Patients in the MPM group also presented more frequently with actinic keratoses (51.1%; *p* = 0.0025) and Non-Melanoma Skin Cancer (NMSC) (38.3%; *p* = 0.01) compared to the other two groups.

### Clinicopathological aspects

Although melanomas developed de novo more frequently in all three groups, in the MPM group the origin was on a previous melanocytic nevus in 34.5% of cases, this percentage being lower in the SM and FM groups (14.2% and 19.7%, respectively) (*p* = 0.0031).

Furthermore, 44.6% of the SM group presented two or more mitoses per field, compared to 26.9% of the FM group and 16.3% of the MPM group (*p* < 0.0001). The presence of moderate-to-intense inflammatory infiltrate was also higher in the SM group (17.9% vs. 13.7% and 14.9%) (*p* = 0.04). A higher percentage of ulcerated melanomas (11.3%) and melanomas with a Breslow thickness greater than 0.8 mm (51.3%) were also found in the SM group compared to the other two groups, although this difference was not statistically significant.

No statistically significant differences were found in terms of melanoma location or histological subtype.

### Personal and family history of cancer and secondary neoplasms

Patients in the FM group had more primary tumors other than melanoma and pancreatic cancer (23 patients; 17.4%) compared to patients in the SM (9.5%) and MPM (6.4%) groups (*p* = 0.03). The most frequent tumors in the FM group were renal cancer (6 cases; 4.5%) and breast cancer (5 cases; 3.8%).

Similarly, first- and second-degree relatives of patients in the FM group also had a higher prevalence of other neoplasms (Table 2). The authors observed significant differences in the prevalence of a family history of breast cancer, with 25% in the FM group, 13.7% in the SM group, and 12.8% in the MPM group (*p* = 0.01). For some tumors, such as colon and ovarian cancer, a higher prevalence was also observed in the FM group (18.9% and 4.5%, respectively), although this did not reach statistical significance when comparing the three groups.

**Table 2:** Prevalence of personal and family history of cancer, with p-values < 0.05 considered statistically significant and shown in bold.

Personal and family history of cancer		Sporadic melanoma	Familial melanoma	Multiple primary melanoma	Total	*p*
		(*n* = 263)	(*n* = 132)	(*n* = 47)	(*n* = 442)	
Personal history of any tumor other than melanoma and pancreas	No	238 (90.5%)	109 (82.6%)	44 (93.6%)	391 (88.5%)	**0.03**
	Yes	25 (9.5%)	23 (17.4%)	3 (6.4%)	51 (11.5%)	
Family history of breast cancer	No	227 (86.3%)	99 (75%)	41 (87.2%)	367 (83%)	**0.01**
	Yes	36 (13.7%)	33 (25%)	6 (12.8%)	75 (17%)	
Family history of lung cancer	No	224 (85.2%)	115 (87.1%)	42 (89.4%)	381 (86.2%)	0.75
	Yes	39 (14.8%)	17 (12.9%)	5 (10.6%)	61 (13.8%)	
Family history of prostate cancer	No	244 (92.8%)	123 (93.2%)	45 (95.7%)	412 (93.2%)	0.89
	Yes	19 (7.2%)	9 (6.8%)	2 (4.3%)	30 (6.8%)	
Family history of kidney cancer	No	259 (98.5%)	129 (97.7%)	46 (97.9%)	434 (98.2%)	0.64
	Yes	4 (1.5%)	3 (2.3%)	1 (2.1%)	8 (1.8%)	
Family history of liver cancer	No	251 (95.4%)	126 (95.5%)	44 (93.6%)	421 (95.2%)	0.80
	Yes	12 (4.6%)	6 (4.5%)	3 (6.4%)	21 (4.8%)	
Family history of colon cancer	No	232 (88.2%)	107 (81.1%)	43 (91.5%)	382 (86.4%)	0.09
	Yes	31 (11.8%)	25 (18.9%)	4 (8.5%)	60 (13.6%)	
Family history of gastric cancer	No	246 (93.5%)	126 (95.5%)	46 (97.9%)	418 (94.6%)	0.46
	Yes	17 (6.5%)	6 (4.5%)	1 (2.1%)	24 (5.4%)	
Family history of bone cancer	No	261 (99.2%)	130 (98.5%)	45 (95.7%)	436 (98.6%)	0.11
	Yes	2 (0.8%)	2 (1.5%)	2 (4.3%)	6 (1.4%)	
Family history of brain cancer	No	258 (98.1%)	130 (98.5%)	46 (97.9%)	434 (98.2%)	1
	Yes	5 (1.9%)	2 (1.5%)	1 (2.1%)	8 (1.8%)	
Family history of ovarian cancer	No	260 (98.9%)	126 (95.5%)	46 (97.9%)	432 (97.7%)	0.08
	Yes	3 (1.1%)	6 (4.5%)	1 (2.1%)	10 (2.3%)	
Family history of endometrial cancer	No	255 (97%)	127 (96.2%)	44 (93.6%)	426 (96.4%)	0.44
	Yes	8 (3%)	5 (3.8%)	3 (6.4%)	16 (3.6%)	
Family history of thyroid cancer	No	260 (98.9%)	130 (98.5%)	47 (100%)	437 (98.9%)	1
	Yes	3 (1.1%)	2 (1.5%)	0 (0%)	5 (1.1%)	
Family history of bladder cancer	No	258 (98.1%)	128 (97%)	45 (95.7%)	431 (97.5%)	0.36
	Yes	5 (1.9%)	4 (3%)	2 (4.3%)	11 (2.5%)	
Family history of hematological neoplasia	No	246 (93.5%)	123 (93.2%)	44 (93.6%)	413 (93.4%)	1
	Yes	17 (6.5%)	9 (6.8%)	3 (6.4%)	29 (6.6%)	
Family history of mesothelioma	No	261 (99.2%)	132 (100%)	47 (100%)	440 (99.5%)	0.64
	Yes	2 (0.8%)	0 (0%)	0 (0%)	2 (0.4%)	

No statistically significant differences were found in the percentage of family history of lung, prostate, kidney, liver, gastric, bone, brain, endometrial, thyroid, bladder, hematological tumors, or mesothelioma cancers. These data are summarized in Table 2.

### Multivariate analysis: characteristics associated with each group

Multivariate analysis confirmed that some characteristics remained as independent associations after adjustment for age, sex, phototype, and other clinical variables.

• MPM: Compared to the other two groups, MPM was significantly associated with the presence of more than 50 melanocytic nevi (OR for fewer than 50 nevi 0.36; 95% CI 0.17–0.72), the presence of actinic keratoses (OR = 3.08; 95% CI 1.40–6.40), fewer than 2 mitoses per field (OR for more than 2 mitoses 0.39; 95% CI 0.15–0.90), and melanoma arising on a melanocytic nevus (OR = 2.45; 95% CI 1.15–5.11). Light hair color showed a trend toward significance in this group (OR for dark hair color 0.61; 95% CI 0.29–1.30; *p* = 0.20). These data are summarized in Table 3 and Fig. 1.

**Table 3:** Multivariate analysis of characteristics associated with multiple primary melanoma, with p-values < 0.05 considered statistically significant and shown in bold.

	Univariate		Multivariate	
	OR (95% CI)	*p*	OR (95% CI)	*p*
Female sex	0.45 (0.23‒0.83)	0.012	‒	‒
Dark eyes	0.44 (0.24‒0.82)	0.01	‒	‒
Dark hair	0.46 (0.25‒0.87)	0.015	0.61 (0.29–1.30)	0.20
Less than 50 nevi	0.36 (0.19‒0.66)	0.001	0.36 (0.17‒0.72)	**0.004**
Presence of actinic keratoses	2.84 (1.53–5.28)	0.001	3.08 (1.40–6.40)	**0.002**
Presence of NMSC	2.36 (1.23–4.44)	0.008	‒	‒
Presence of 2 or more mitoses	0.30 (0.12‒0.67)	0.006	0.39 (0.15‒0.90)	**0.038**
Melanoma arising on melanocytic nevus	2.80 (1.41–5.41)	0.002	2.45 (1.15–5.11)	**0.017**

**Fig. 1:**
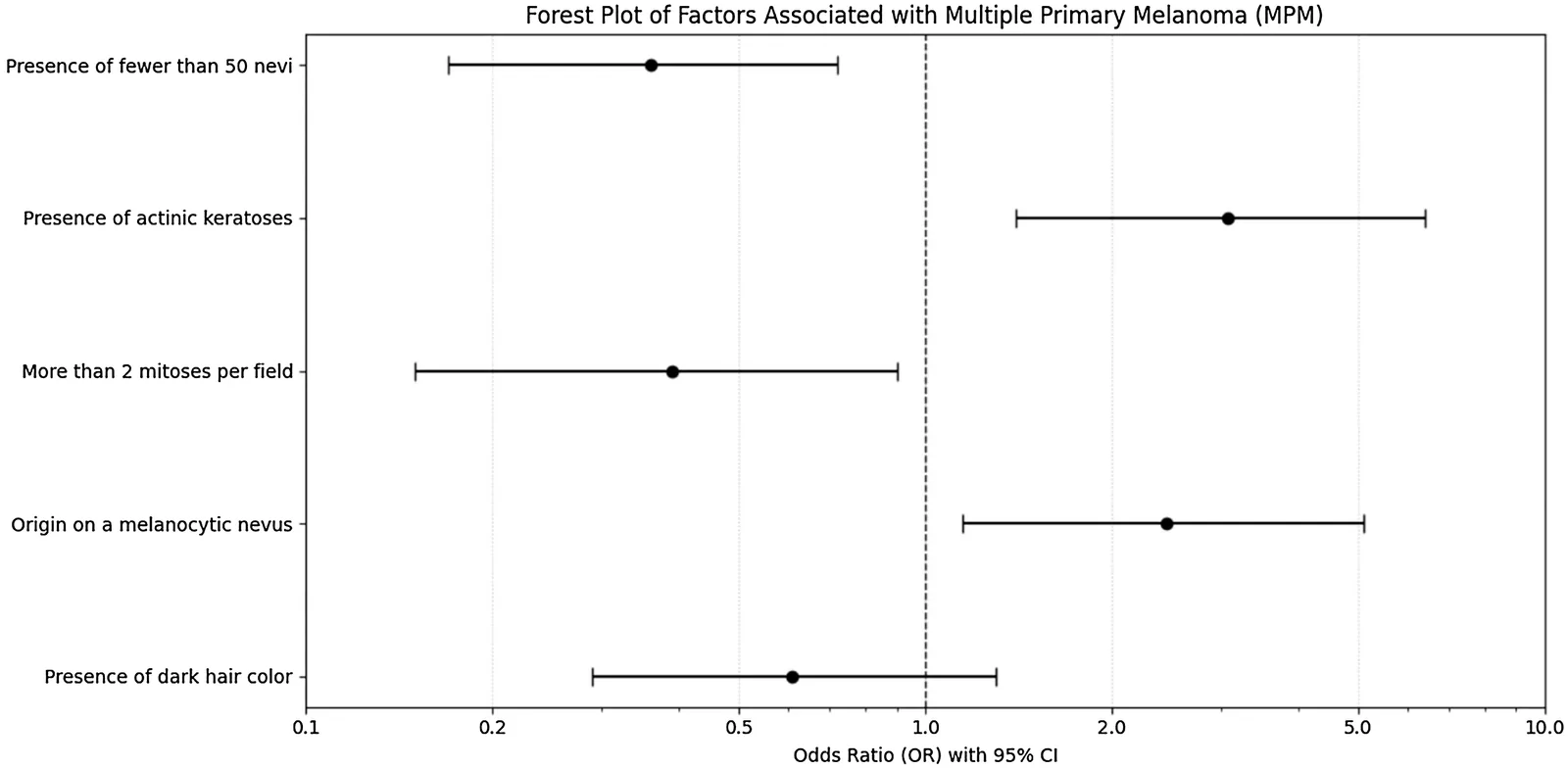
Factors associated with multiple primary melanoma, with odds ratios and 95% confidence intervals obtained from the multivariable logistic regression model.
• FM: Compared to the other groups, FM showed an independent association with age under 45-years (OR for age over 45-years 0.52; 95% CI 0.33‒0.83), absence of ulceration (OR for presence of ulceration 0.31; 95% CI 0.09‒0.79), and a history of neoplasia other than melanoma and pancreatic cancer (OR = 2.52; 95% CI 1.33–4.78). Among the oncological family history, breast cancer (OR = 2.17; 95% CI 1.25–3.76) and ovarian cancer (OR = 4.94; 95% CI 1.32–20.91) were associated. A non-significant trend was observed for family history of colon cancer (OR = 1.65; 95% CI 0.90–2.98). These data are summarized in Table 4 and Fig. 2.

**Table 4:** Multivariate analysis of characteristics associated with familial melanoma, with p-values < 0.05 considered statistically significant and shown in bold.

Variables	Univariate		Multivariate	
	OR (95% CI)	*p*	OR (95% CI)	*p*
Female sex	1.50 (0.99–2.3)	0.054	‒	‒
Dark eyes	1.56 (1.01–2.44)	0.047	‒	‒
Age over 45 years	0.52 (0.34‒0.80)	0.003	0.52 (0.33‒0.83)	**0.005**
Breslow greater than 0.8 mm	0.64 (0.42‒0.97)	0.038	‒	‒
Presence of 2 or more mitoses	0.54 (0.33‒0.86)	0.011	‒	‒
Presence of ulceration	0.34 (0.11‒0.82)	0.029	0.31 (0.09‒0.79)	**0.025**
Personal history of neoplasia	2.12 (1.16–3.84)	0.013	2.52 (1.33–4.78)	**0.004**
Family history of breast cancer	2.12 (1.27–3.54)	0.004	2.17 (1.25–3.76)	**0.005**
Family history of colon cancer	1.83 (1.04–3.20)	0.033	1.65 (0.90–2.98)	0.099
Family history of ovarian cancer	3.64 (1.02–14.45)	0.048	4.94 (1.32–20.91)	**0.019**

**Fig. 2:**
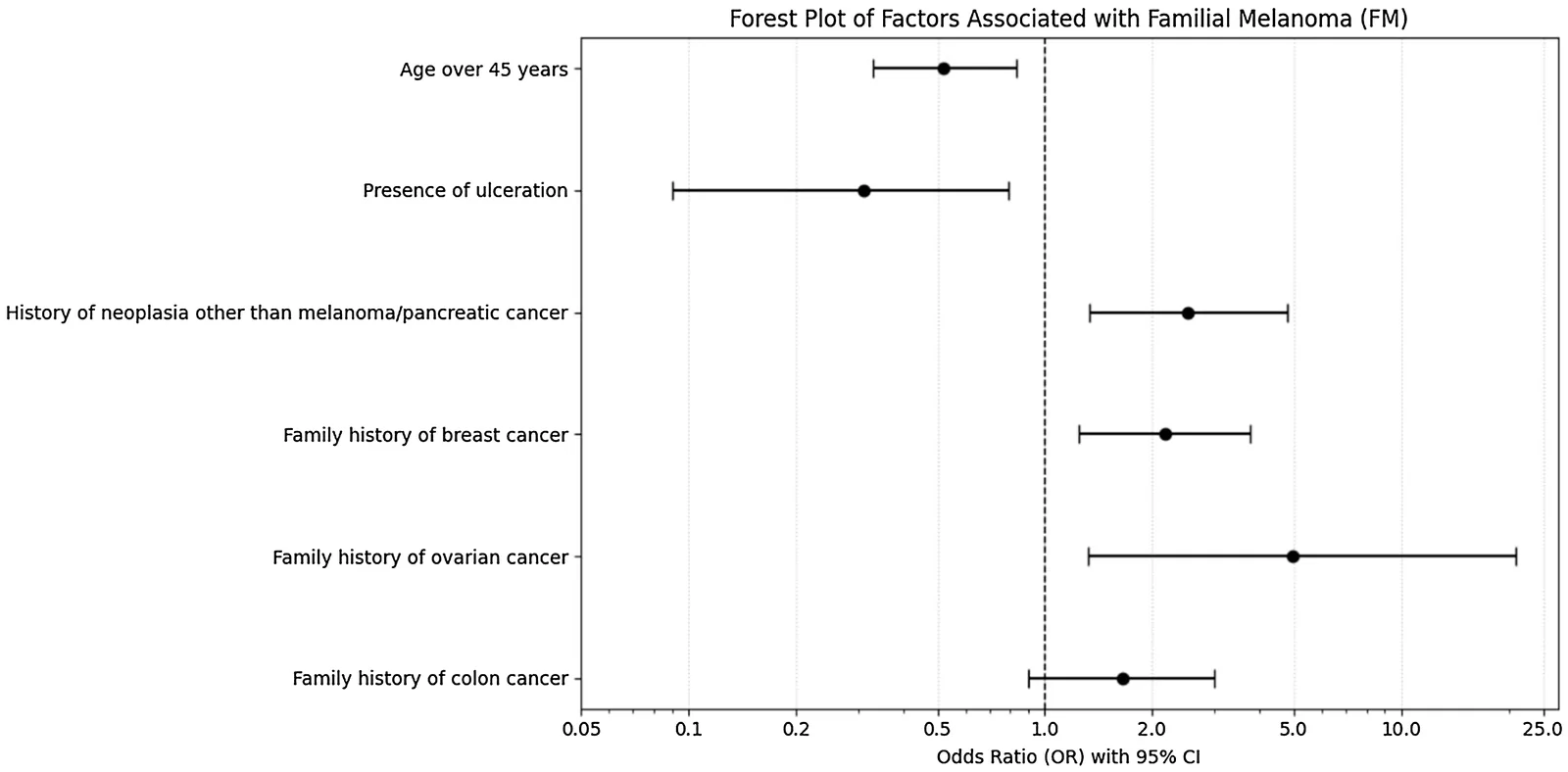
Factors associated with familial melanoma, with odds ratios and 95% confidence intervals obtained from the multivariable logistic regression model.
• SM: The presence of fewer than 50 melanocytic nevi (OR for more than 50 nevi 0.47; 95% CI 0.30–0.74), absence of non-melanoma skin cancer (OR for presence of non-melanoma skin cancer 0.58; 95% CI 0.35–0.97), and the presence of 2 or more mitoses (OR = 1.82; 95% CI 1.13–2.97) were independently associated with SM. The presence of ulceration (OR = 1.76; 95% CI 0.76–4.48) and de novo melanoma (OR for origin on a melanocytic nevus 0.61; 95% CI 0.36–1.05) showed a trend toward significance in the SM group. These data are summarized in Table 5 and Fig. 3.

**Table 5:** Multivariate analysis of characteristics associated with sporadic melanoma, with p-values < 0.05 considered statistically significant and shown in bold.

Variables	Univariate		Mulvariate	
	OR (95% CI)	*p*	OR (95% CI)	*p*
More than 50 nevi	0.47 (0.31‒0.71)	<0.001	0.47 (0.30‒0.74)	**0.001**
Presence of NMSC	0.60 (0.38‒0.94)	0.026	0.58 (0.35‒0.97)	**0.038**
Presence of 2 or more mitoses	2.51 (1.62–3.94)	<0.001	1.82 (1.13–2.97)	**0.014**
Presence of ulceration	2.23 (1.06–5.12)	0.042	1.76 (0.76–4.48)	0.202
Melanoma arising on melanocytic nevus	0.52 (0.31‒0.86)	0.011	0.61 (0.36–1.05)	0.07
Breslow greater than 0.8 mm	1.56 (1.06–2.31)	0.023	‒	‒
Moderate-to-intense inflammatory infiltrate	1.36 (0.80–2.34)	0.258	‒	‒

**Fig. 3:**
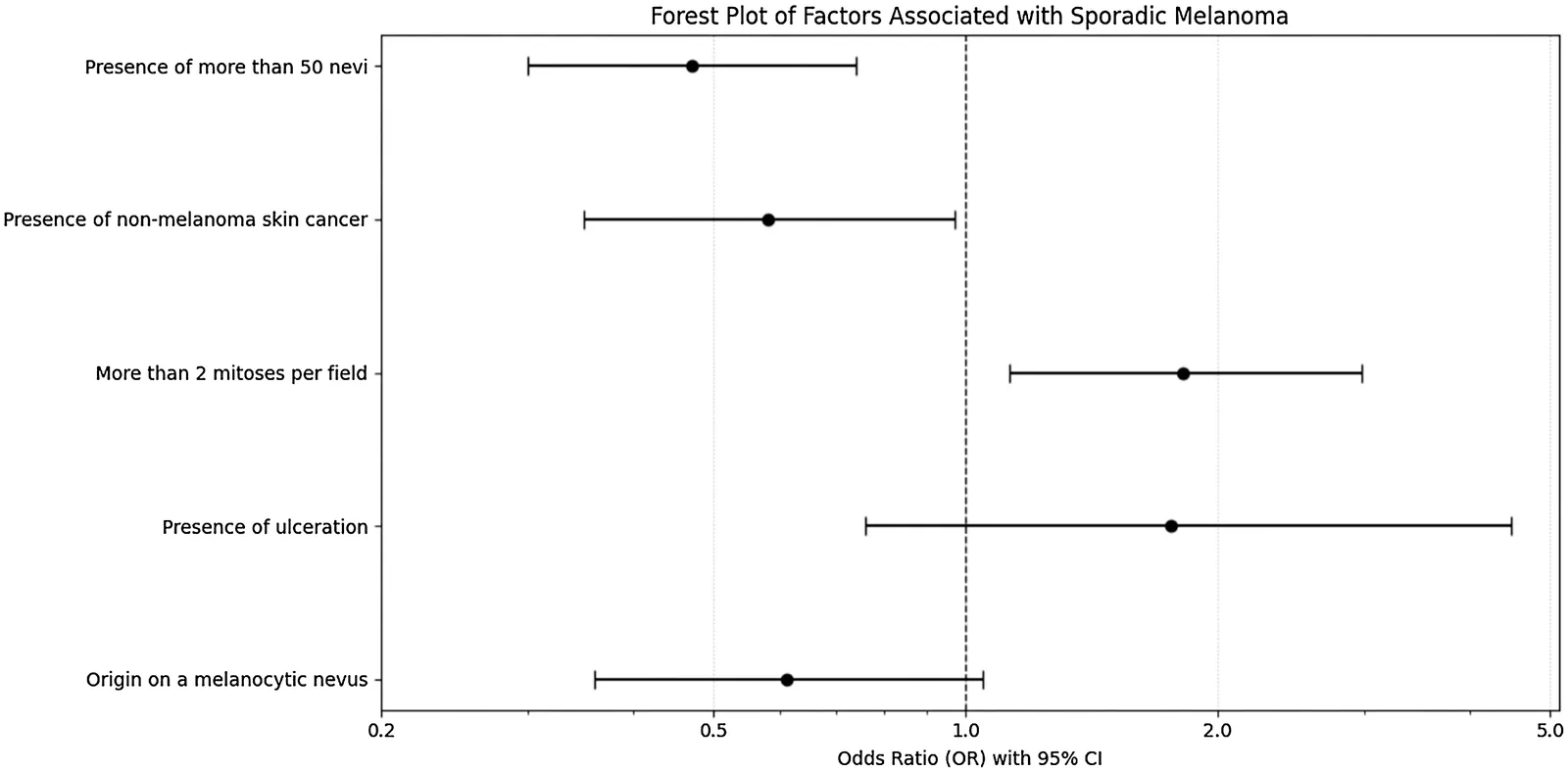
Factors associated with sporadic melanoma, with odds ratios and 95% confidence intervals obtained from the multivariable logistic regression model.

## Discussion

The authors observed differences in phenotypic, clinicopathological, and epidemiological characteristics between the three groups. After multivariate analysis, the authors continued to observe statistically significant differences.

In the present series, the MPM group presents low phototypes, with light eyes and hair, and more than 50 melanocytic nevi. In a meta-analysis, fair hair and light eye color were significantly associated with MPM, with an OR of 1.44, as well as male sex, older age and inadequate sun protection. The presence of dysplastic nevi, which are more common in fair-skinned individuals, was also correlated with a higher risk of MPM.^17^ The authors have observed a male predominance in this group similar to that reported in the literature.^17^, ^18^ In the present series, patients with MPM also frequently developed melanoma arising from melanocytic nevi. This finding has been associated in the literature with fair skin type, the presence of multiple nevi, and the absence of a family history of melanoma.^19^

Patients with MPM also showed a higher prevalence of actinic keratoses, which are considered a marker of chronic sun damage and precursor lesions of NMSC. Their presence has been shown to multiply the risk of developing NMSC, but also melanoma, with a risk up to 2.7-times higher than in the general population.^20^ This data support the hypothesis that in the MPM group, light phototype, accumulated sun damage, and a constitutional susceptibility to melanocytic proliferation could favor the appearance of multiple melanomas.

In contrast, in the FM group, the authors observed a higher percentage of patients under 45-years of age and an absence of signs of chronic sun damage, and they characteristically presented with thinner, non-ulcerated melanomas. It has been previously noted that patients with FM develop melanomas with lower Breslow thickness and a higher rate of melanoma in situ.^13^, ^21^ Most authors also agree that patients with a family history of melanoma are usually diagnosed at younger ages.^6^, ^22^ It seems reasonable to think that patients with FM are characterized by a strong hereditary predisposition, consistent with what has been described in the literature, which highlights germline mutations in susceptibility genes such as CDKN2A. The lower frequency of ulceration observed in this group could reflect earlier detection in the context of family follow-up, or a different tumor biology.

In FM, the authors have observed a high burden of personal and family history of cancer beyond melanoma and pancreatic cancer. Among family history, the high prevalence of breast cancer stands out, and to a lesser extent, ovarian cancer. Previous studies have described the association of CDKN2A with breast cancer;^23^ cases of breast cancer in patients with a BAP1 mutation^24^ and a higher prevalence of melanoma in patients with a BRCA2 mutation.^25^, ^26^, ^27^ These data support expanding the criteria for genetic testing in individuals under 45-years of age, those with a history of a second neoplasm, and those with a family history of breast/ovarian cancer.^14^, ^16^

In accordance with other studies, patients with SM present with few nevi and aggressive, ulcerated melanomas with a higher number of mitoses.^12^, ^13^, ^28^ These patients have not typically been followed as closely prior to diagnosis, which could partly explain the higher Breslow thickness.^13^ In the present series, patients in the SM group presented less frequently with signs of chronic sun damage (actinic keratoses and NMSCs) compared to the MPM group. In previous studies, SM patients frequently presented with actinic keratoses and multiple solar lentigines.^12^ However, most studies compared patients with one or more melanomas without family history with patients who met the criteria for familial melanoma, without distinguishing between SM and MPM.^12^, ^13^, ^28^

In this regard, it seems likely that other underlying mechanisms exist for the development of SM. This subtype of melanoma could represent a different biological entity, not explained by cumulative sun damage or hereditary predisposition, but possibly by sporadic somatic mutations, intermittent sun exposure, or immunological alterations.

A few limitations should be noted. It is possible that some patients develop a second melanoma in the future, so long term follow-up for secondary melanoma is needed for validation. Also certain tumors, such as pancreatic and ovarian cancer, are probably underrepresented in this series due to their high mortality rate. Furthermore, some participants may not recall or be aware of certain family history.

## Conclusion

The three subgroups present distinct clinical, histological, and epidemiological profiles.

Patients with MPM present with fair skin types, more than 50 nevi, and signs of chronic sun damage. In this group, melanoma is frequently found arising on melanocytic nevi. All of this points to a central role of phototype and cumulative sun exposure.

Patients with SM are younger and develop non-ulcerated melanomas with thin Breslow thickness, and a higher burden of personal and family cancers, such as breast and ovarian cancer. This suggests a genetic predisposition to cancer in these patients, supporting the expansion of genetic testing indications considering age and other cancer history.

Finally, patients with SM present with few nevi and aggressive melanomas (ulcerated and with high mitotic rates), and less evidence of photodamage. This subtype of melanoma could represent a different biological entity, unrelated to chronic sun damage or hereditary predisposition.

This study highlights the complexity of melanoma risk factors, suggesting that both genetic and environmental elements play a critical role in its development. Larger studies would be interesting for further investigation, to optimize identification, prevention and prognosis in each subgroup.

## Authors' contributions

Irene López Riquelme: The study concept and design; data collection, or analysis and interpretation of data; statistical analysis; writing of the manuscript or critical review of important intellectual content; intellectual participation in the propaedeutic and/or therapeutic conduct of the studied cases; critical review of the literature; final approval of the final version of the manuscript.

Silvestre Martínez García: The study concept and design; data collection, or analysis and interpretation of data; effective participation in the research guidance; intellectual participation in the propaedeutic and/or therapeutic conduct of the studied cases; final approval of the final version of the manuscript.

Marina Romero Bravo: Data collection, or analysis and interpretation of data; final approval of the final version of the manuscript.

Miguel Ángel Berciano Guerrero: The study concept and design; effective participation in the research guidance; intellectual participation in the propaedeutic and/or therapeutic conduct of the studied cases; final approval of the final version of the manuscript.

## Ethical considerations

This study was approved by the Provincial Research Ethics Committee of Malaga on May 29, 2025. Research Code: SICEIA-2025-001211.

## Declaration of Generative AI and AI-assisted technologies in the writing process

During the preparation of this work, the authors used SCISPACE in order to generate a forest plot to represent multivariate analysis results. After using this tool, the authors reviewed and edited the content as needed and take full responsibility for the content of the published article.

## Financial support

None declared.

## Research data availability

The entire dataset supporting the results of this study was published in this article.

## Conflicts of interest

None declared.
