# Supplementary material for: Clinical, phenotypic and oncologic background differences in patients with sporadic, familial and multiple primary melanoma
Source: An Bras Dermatol. 2026 Oct 31;101(6):501472. doi: 10.1016/j.abd.2026.501472 (PMC13744214; doi:10.1016/j.abd.2026.501472)
Supplement: Supplementary file 1 [file mmc1.docx]

**ABD-D-26-00319**

**Supplementary Material** STROBE Checklist.

STROBE Checklist – Cross-Sectional Study

Observational study including 442 patients with melanoma.

| **Item** | **Recommendation** | **Section / Page** |
| --- | --- | --- |
| 1a | Design of the study in title or abstract | Abstract / Page 1 |
| 1b | Informative and balanced summary | Abstract / Page 1 |
| 2 | Background | Introduction / Page 3 |
| 3 | Specific objective | Introduction / Page 4 |
| 4 | Key elements of study design | Methods – Study design/ Page 5 |
| 5 | Setting, locations, and relevant dates | Methods – Setting / Page 5 |
| 6a | Eligibility criteria | Methods – Participants / Page 5 |
| 7 | Outcomes, exposures, predictors, potential confounders, and effect modifiers | Methods – Variables / Page 5 |
| 8 | Data sources/ measurement | Methods – Variables / Proceedings / Page 5 |
| 9 | Potential sources of bias | Methods and Discussion – Bias / Limitations / Pages 5 and 11 |
| 10 | Sample size | Methods – Sample size / Page 5 |
| 11 | Quantitative variables | Methods – Statistical analysis / Page 6 |
| 12a | Statistical methods | Methods – Statistical analysis / Page 6 |
| 12b | Subgroups | Methods and Results / Pages 5 and 6 |
| 12c | Missing data | Methods– Statistical analysis / Page 5 |
| 13a | Number of participants | Results / Page 6 |
| 14a | Characteristics of study participants | Results / Pages 6 to 8 – Table 1 |
| 14b | Missing data of variables | Results / Pages 6‒8 |
| 15 | Results data | Results / Pages 6‒9 |
| 16a | Unadjusted estimates and confounder-adjusted estimates | Results / Pages 6‒9 and Tables 1‒5 |
| 16b | Category boundaries when continuous variables are categorized | Methods /page 5 – Variables / Results /page 6‒8 / Tables 1 and 2 |
| 17 | Other analysis (subgroups, sensitivity) | Results / pages 8‒9 |
| 18 | Summary of key results | Discussion – Interpretation /pages 9‒11 |
| 19 | Study limitations | Discussion and Conclusions– Limitations /pages 11 and 12 |
| 20 | Overall interpretation | Discussion / pages 9‒11 |
| 21 | Generalisability | Discussion – generalisability / page 9‒11 |
| 22 | Source of funding | Title page – Funding |
